# Axonal Growth and Fasciculation of Spinal Neurons Promoted by Aldynoglia in Alkaline Fibrin Hydrogel: Influence of Tol-51 Sulfoglycolipid

**DOI:** 10.3390/ijms25179173

**Published:** 2024-08-23

**Authors:** Vinnitsa Buzoianu-Anguiano, Alejandro Arriero-Cabañero, Alfonso Fernández-Mayoralas, Mabel Torres-Llacsa, Ernesto Doncel-Pérez

**Affiliations:** 1 Hospital Nacional de Parapléjicos, Servicio de Salud de Castilla La Mancha, 45071 Toledo, Spain; vbuzoianu@externas.sescam.jccm.es (V.B.-A.); aarrieroc@externas.sescam.jccm.es (A.A.-C.); mabelt@sescam.jccm.es (M.T.-L.); 2 Departamento de Química Bio-Orgánica, Instituto de Química Orgánica General (IQOG-CSIC), CSIC, 28006 Madrid, Spain; mayoralas@iqog.csic.es

**Keywords:** aldynoglia, alkaline fibrin, dorsal root ganglia, spinal neurons, Tol-51 sulfoglycolipid, traumatic spinal cord injury

## Abstract

Traumatic spinal cord injury (tSCI) has complex pathophysiological events that begin after the initial trauma. One such event is fibroglial scar formation by fibroblasts and reactive astrocytes. A strong inhibition of axonal growth is caused by the activated astroglial cells as a component of fibroglial scarring through the production of inhibitory molecules, such as chondroitin sulfate proteoglycans or myelin-associated proteins. Here, we used neural precursor cells (aldynoglia) as promoters of axonal growth and a fibrin hydrogel gelled under alkaline conditions to support and guide neuronal cell growth, respectively. We added Tol-51 sulfoglycolipid as a synthetic inhibitor of astrocyte and microglia in order to test its effect on the axonal growth-promoting function of aldynoglia precursor cells. We obtained an increase in GFAP expression corresponding to the expected glial phenotype for aldynoglia cells cultured in alkaline fibrin. In co-cultures of dorsal root ganglia (DRG) and aldynoglia, the axonal growth promotion of DRG neurons by aldynoglia was not affected. We observed that the neural precursor cells first clustered together and then formed niches from which aldynoglia cells grew and connected to groups of adjacent cells. We conclude that the combination of alkaline fibrin with synthetic sulfoglycolipid Tol-51 increased cell adhesion, cell migration, fasciculation, and axonal growth capacity, promoted by aldynoglia cells. There was no negative effect on the behavior of aldynoglia cells after the addition of sulfoglycolipid Tol-51, suggesting that a combination of aldynoglia plus alkaline fibrin and Tol-51 compound could be useful as a therapeutic strategy for tSCI repair.

## 1. Introduction

Traumatic spinal cord injury (tSCI) refers to a sudden, external physical impact (from a traffic accident, fall, sports-related injury, or violent attack) that severely damages the spinal cord [1]. tSCI is a complex pathology that causes loss of sensory and motor functions. Additionally, tSCI is a continuous process resulting from initial mechanical injury and subsequent vascular, tissular, and cellular changes. Two critical events are produced: the primary injury caused by mechanical damage, and the secondary injury that triggers different mechanisms of cell death. The secondary injury occurs in three phases: acute phase (0–2 days), subacute phase (3 days to weeks), and chronic phase (7 weeks to years) after SCI. In the acute and subacute phases, there is excitotoxicity by glutamate lipoperoxidation, an increasing immune response, and initiation of the formation of a fibroglial scar. In the chronic phase, the maturation of the fibroglial scar formation continues, along with demyelination and neurodegeneration processes [2,3,4]

As mentioned above, the pathophysiology of tSCI is a continuum that begins with a primary injury caused by mechanical trauma. It originates by compression, stretching, laceration, or transection of the spinal cord, causing hemorrhage, alteration to the blood–brain barrier, and consequently rupture of axons, rupture of membranes, and death of neurons, glia, and endothelial cells. As part of a continuum, the primary lesion leads to the pathophysiology events of the secondary injury phase, characterized by edema, hypoxia and ischemia, excitotoxicity, electrolyte changes, production of free radicals, inflammation, and multiple other alterations, resulting in a prolonged period of destruction of spinal cord tissue [5]

**Figure 1:**
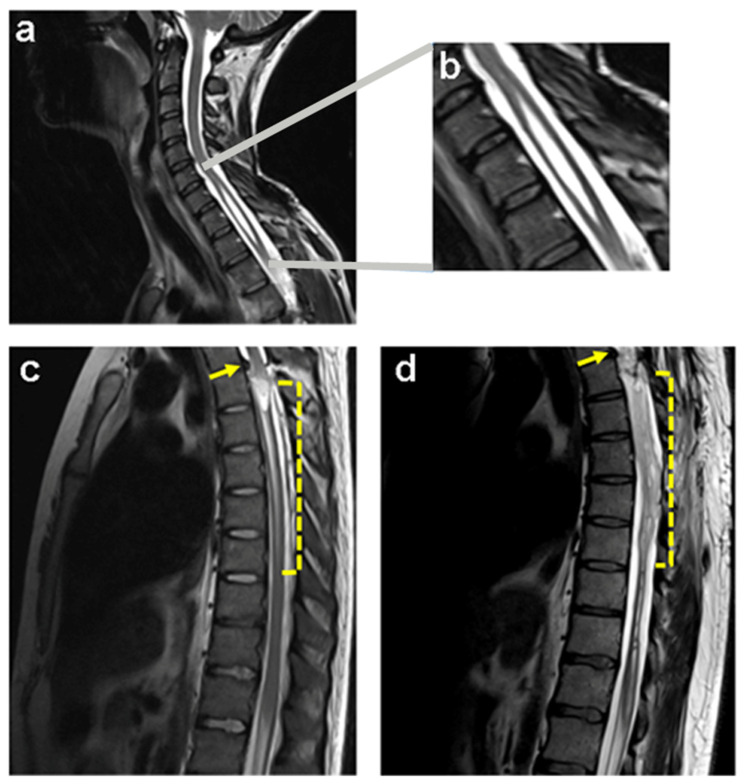
Subacute and chronic lesions of the spinal cord in human patients observed by magnetic resonance imaging. Syringomyelia cyst in a child due to idiopathic causes (**a**) shows the enlargement of the cavity within the spinal cord (**b**). The dotted areas (**c**,**d**) indicate a decade of evolution in a patient from subacute injury (**c**) to chronic spinal injury (**d**) produced by traumatic disk herniation at the second to third vertebral thoracic levels, T2–T3 (see arrows). Note the formation of syringomyelia below the lesion at the beginning (**c**) and how ten years later, the spinal cord becomes necrotic (**d**).

A clear example of neural degeneration in tSCI is syringomyelia, which occurs frequently in patients with complete SCI within 5 years after injury [6]. The devastating course of syringomyelia leads to the destruction of the parenchymal tissue of the spinal cord and the formation of a cavity during the transition from the subacute to the chronic stage of SCI (Figure 1).

To address loss of spinal tissue, fill the cavity, and restore axonal communication, the use of hydrogels and cells has been proposed [7]. Fibrin is a hydrogel with a strand structure, thus creating a continuous network immersed in a fluid, giving rise to adhesion sites for cells and facilitating axonal migration [8]. This is the main component of the blood clot and acts as a support base for cell attachment and cell–cell interaction [9]. Fibrin hydrogels provide growth-permissive substrates and serve as carriers for therapeutic cell transplantation into an injured spinal cord. However, the application of fibrin hydrogels may be limited due to their relatively rapid degradation rate in vivo [10]. Here, we used an alkaline-modified fibrin gel as a cell substrate that maintains some of the adhesive properties of the original fibrin and facilitates cell migration.

Aldynoglia are similar to the Schwann cells of the peripheral nervous system, but they are glial neural precursor cells (NPCs) of the central nervous system (CNS) grouped by their common functions and immunophenotypic markers [2,11,12]. Their proliferative and growth-promoting capacity is maintained in the adult CNS with functions specific to the site in which they are found [13]. The functions of macroglial types, such as tanycytes, pituicytes, and olfactory ensheathing cells, involves promoting growth of axons and their rapid, reversible wrapping. Aldynoglia, like Schwann cells, express soluble and membrane-bound molecules capable of promoting neurite outgrowth. There is a high neurite growth-promoting ability of neurosphere-derived aldynoglia that promotes interneurite association to form longer and thicker fascicles [12,14,15]. In this work, we used aldynoglia cells derived from embryonic striatum to test the promotion of axonal growth and fasciculation of spinal neurons.

Axonal growth inhibitors are released after tSCI. They accumulate at the site of the injury and form a highly inhibitory environment for axonal regeneration. The inhibitory effect is produced by the binding of inhibitory molecules (myelin-associated inhibitors, chondroitin sulfate proteoglycans, etc.) to specific receptors located on the membranes of neurons (for example, the Nogo 1 receptor, the leucine-rich repeat and Ig domain-containing 1, the neurotrophin receptor p75, etc.). Upon activation, these receptors initiate downstream signaling pathways, where RhoA/ROCK signaling is the most relevant pathway [16].

An additional approach for tSCI therapy is to use a Rho GTPase inhibitor to disrupt the RhoA/ROCK cascade and consequently halt the collapse of axonal growth [15]. A sulfoglycolipid compound, formerly called IG20 and now renamed Tol-51, is able to inhibit GFAP+ astroglial cells while increasing the number of cells expressing neurofilament and myelin, demonstrating that the synthetic sulfoglycolipid can increase neuronal activity and improve locomotor recovery after tSCI in rats with spinal contusion [17].

This study evaluates the efficacy of the simultaneous combination of alkaline fibrin, aldynoglia, and Tol-51 in 3D cultures of neural cells. Cellular properties relevant in tSCI restoration, such as cell migration, neural cell differentiation, axonal growth capacity, and axonal fasciculation of spinal neurons, were monitored under in vitro conditions. We finally arrived at an optimized combination in which these new therapeutic elements promoted axonal growth and fasciculation of axons. This combinatorial approach could form the basis of a new treatment for tSCI condition.

## 2. Results

### 2.1. Alkaline Fibrin Hydrogel and Tol-51 Sulfoglycolipid

**Figure 2:**
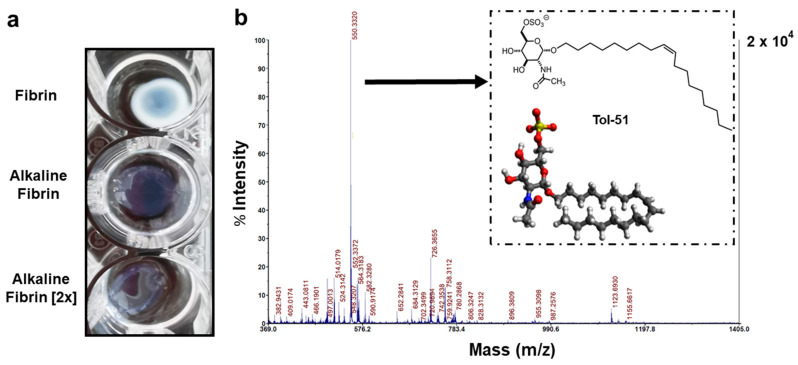
Alkaline conditions for fibrin hydrogel gelation and Tol-51 sulfoglycolipid mass spectrum with molecular structures. (**a**) Fibrin hydrogels 1 h after gelation in physiological and alkaline conditions, with normal or double (2×) concentration of TBS ingredients. (**b**) Signal at 550.33 in the negative ion mode mass spectrum for the sulfoglycolipid Tol-51. In the box, the carbon structure and the ball-and-stick model of the Tol-51 molecule are depicted at top and bottom, respectively. Note the greater transparency in the fibrin hydrogel under alkaline conditions (**a**) and the folding possibility in aliphatic moiety for Tol-51 compound (**b**).

We previously described a method to obtain alkaline hydrogel suitable for oriented cell growth of glial cells [18]. This time, we used different concentrations of TBS (pH = 10.0) to dilute the fibrinogen component of Tisseel, a fibrin pharmaceutical, in order to produce an alkaline fibrin. The gelation of fibrin in the presence of TBS produced a translucent hydrogel that contrasts with a hyaline fibrin performed under neutral pH conditions. Two concentrations of TBS ingredients were used to make two types of fibrin at basic pH. Both alkaline fibrin hydrogels showed lower gel consistency but higher transparency and laxity compared to the firmly gelled fibrin used as a control (Figure 2a).

In previous reports, we described the organic synthesis of compound 51 (C26H48KNO9S; MW: 589.82) with growth-inhibitory activity in human melanoma or glioma cells [19]). We later named this compound IG20 as a promoter of axonal growth and myelin production for neurons and oligodendrocytes, respectively [17,20]. We now change the name of this sulfoglycolipid compound to Tol-51 to avoid misunderstandings with other reported molecules, and to indicate the use of the Tol-51 compound in traumatic spinal cord injury (tSCI) research in Toledo at the National Hospital for Paraplegics (Figure 2b).

The presence of sulfoglycolipid Tol-51 in cell culture media was monitored by mass spectrometry. The expected signal at 550.33 *m*/*z* was obtained using the negative ion mode, this signal corresponding to the sulfoglycolipid Tol-51. The molecular structure of the Tol-51 compound is represented by the carbon structure and the ball-and-stick model. The foldability of the aliphatic moiety is represented from the extended to the folded form in carbon structure and ball-and-stick models, respectively (Figure 2b).

### 2.2. Differentiation to Aldynoglia Phenotype of Neural Precursor Cells in Alkaline Fibrin Hydrogels

**Figure 3:**
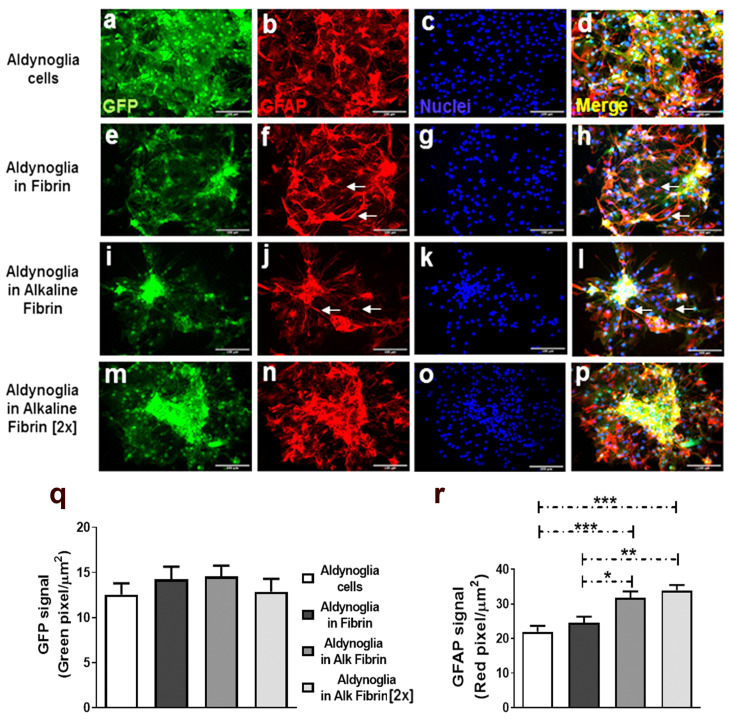
Increasing expression of GFAP after differentiation of neural precursor cells to aldynoglia phenotype in alkaline fibrin hydrogels. After one week in cell culture, aldynoglia–GFP cells in fibrin hydrogels made clusters (**e**,**i**,**m**) in the GFP signaling areas, but not in control cells (**a**). No significant differences in constitutive GFP expression were obtained in areas for cell differentiation analysis (**q**). However, in these GFP+ areas, the alkaline fibrin variants had significantly higher GFAP expression (**j**,**n**) than controls (**b**,**f**), as shown in (**r**). The white arrows point to GFAP bridges between clusters of aldynoglia. Nuclei revealed by Hoechst agent (**c**,**g**,**k**,**o**) and merging zones of GFP, GFAP signals (**d**,**h**,**l**,**p**) are shown One-way ANOVA followed by Tukey’s test were used in statistical analysis (* *p* = 0.01, ** *p* = 0.001 and *** *p* = 0.0001); magnification bars at 500 µm.

Cell differentiation was followed in fixed cell cultures by fluorescence density analysis for green fluorescent protein (GFP) and glial fibrillary acidic protein (GFAP) to distinguish total cells from differentiated glial cells, respectively. After one week, GFP neural precursor cells (GFP-NPCs) differentiated into GFP aldynoglia in fibrin hydrogels and alkaline fibrin hydrogels (Figure 3e–p). NPCs that were cultured under two-dimensional conditions were used as control for the aldynoglia cell differentiation process (Figure 3a–d). No significant differences were obtained for GFP signal expression area, inferring that the number of cells did not increase under the conditions tested, as expected for the differentiation process of aldynoglia cells (Figure 3q). However, the GFAP signal expression area was significantly higher in cells cultured on fibrin hydrogels than in 2D control cells based on one-way ANOVA followed by Tukey’s test; *p* = 0.0001. We also observed that GFP aldynoglia cells formed clusters with significantly higher GFAP expression in alkaline fibrin hydrogels (*p* ≤ 0.01) than cells in control fibrin. These groups of aldynoglia cells were more frequently connected through several long GFAP-positive glial processes in neutral pH fibrin or slightly alkaline fibrin (Figure 3f,h,j,l) than in higher pH conditions, like alkaline fibrin (2×) (Figure 3).

### 2.3. Alkaline Fibrin and Tol-51 Increase GFAP Expression in Aldynoglia

**Figure 4:**
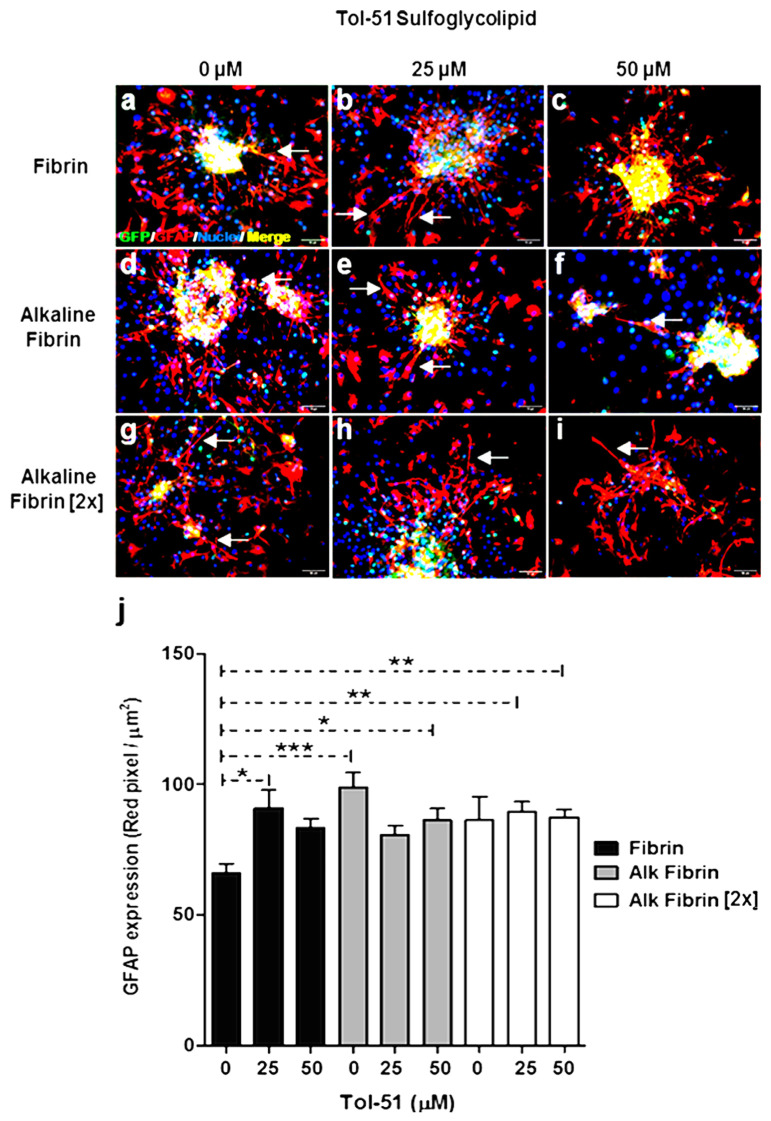
GFAP expression for aldynoglia cultured in alkaline fibrins in the presence of sulfoglycolipid Tol-51. Significant differences in the areas of GFAP expression were obtained in the presence of alkaline fibrins and sulfoglycolipid Tol-51 versus control fibrin, but no additive effect was observed (**j**). Less aggregation of aldynoglia–GFP cells (**g**–**i**) was observed in alkaline (2×) fibrin than in other fibrin hydrogels (**a**–**f**). White arrows point to some of the GFAP glial process that linked aldynoglia cell clusters inside hydrogels (**d**,**f**). Saturation zones (white) were not considered for GFAP expression evaluation. One-way ANOVA followed by Kruskal–Wallis test: * *p* = 0.05, ** *p* = 0.005 and *** *p* = 0.0005; magnification bars at 100 µm.

The significant increase in GFAP marker expression observed in GFP–aldynoglia cells by culture in alkaline fibrin variants was not changed by the addition of Tol-51 sulfoglycolipid (0, 25, and 50 μM). However, significant differences were obtained by the addition of Tol-51 compared to untreated cells in control fibrin based on one-way ANOVA followed by Kruskal–Wallis test; *p* ≤ 0.05 (Figure 4j). Then, alkaline fibrin and Tol-51 produced similar increase in GFAP expression by aldynoglia cells, whether present individually or together (Figure 4).

Clusters of GFP–aldynoglia cells were more compacted in the control fibrin and alkaline fibrin gels (Figure 4a–f) than in concentrated alkaline fibrin variant (2×); (Figure 4g–i). This cell regrouping behavior in cell cultures occurs in aldynoglia precursor cells [12] and is why we selected the alkaline fibrin condition for subsequent assays. The presence of a long GFAP+ process was frequently observed in tested conditions (see arrows in Figure 3 and Figure 4), and this could be useful in tSCI therapy, because GFAP-positive tissue bridges at the spinal cord injury site served as connection for axons [21]. Here, the GFAP+ glial process eventually connected GFP–aldynoglia clusters (Figure 4).

### 2.4. The Interaction of GFP–Aldynoglia Cells and DRG Explant in Alkaline Fibrin Hydrogel Favored Cell Migration

**Figure 5:**
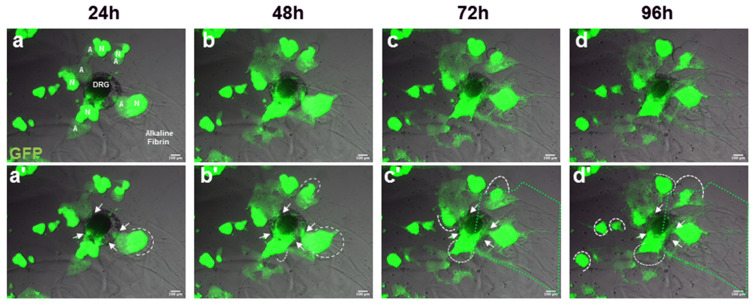
Dynamic cellular interaction of aldynoglia–GFP cells and DRG explants in alkaline fibrin hydrogel. Cell cultures was recorded by time-lapse video up to 96 h, and profuse migration of aldynoglia–GFP cells to DRGs was observed in this period (**a**–**d**). Differentiation of neurospheres into aldynoglia inside alkaline fibrin began at approximately 24 h in cell culture (**a**). Aldynoglia–GFP interaction with DRG explant, fibrin degradation, and cell migration are indicated (**a’**–**d’**). Alkaline fibrin degradation and massive cellular invasion of DRGs by aldynoglia–GFP cells are denoted by white dotted lines and arrows, respectively (**a’**–**d’**). On the third and fourth day (**c**,**d**), large areas of cell migration with aldynoglia–GFP and ganglion cells were also observed throughout the alkaline fibrin matrix—see green dotted area (**c’**,**d’**). A, aldynoglia; DRG, dorsal root ganglion; N, neurosphere; arrowheads, aldynoglia–GFP and DRG cell contact site; white dotted line, zones of alkaline fibrin hydrogel degradation by aldynoglia–GFP cells; green dotted area, cell migration zone in alkaline fibrin.

Aldynoglia cells have a high cell migration capacity under in vitro conditions; and co-culture of dorsal root ganglia (DRGs) with aldynoglia favors ensheathment, premyelination, and axonal growth of spinal neurons [12]. We cultured DRGs with neurosphere-GFP in alkaline fibrin, and these co-cultures were recorded for 4 days by time-lapse microscopy. Cellular differentiation from neurosphere-GFP neural precursor cells to cells with the appearance of GFP–aldynoglia cells was observed at 24 h. The invasion of DRGs and the degradation of alkaline fibrin by GFP–aldynoglia were concomitant events during cell culture that ultimately favored cell migration of individualized GFP–aldynoglia and DRG cells inside the fibrin hydrogel (Figure 5).

### 2.5. Promotion of Axonal Growth of Spinal Neurons by Aldynoglia Is Not Affected by Alkaline Fibrin or the Sulfoglycolipid Tol-51

**Figure 6:**
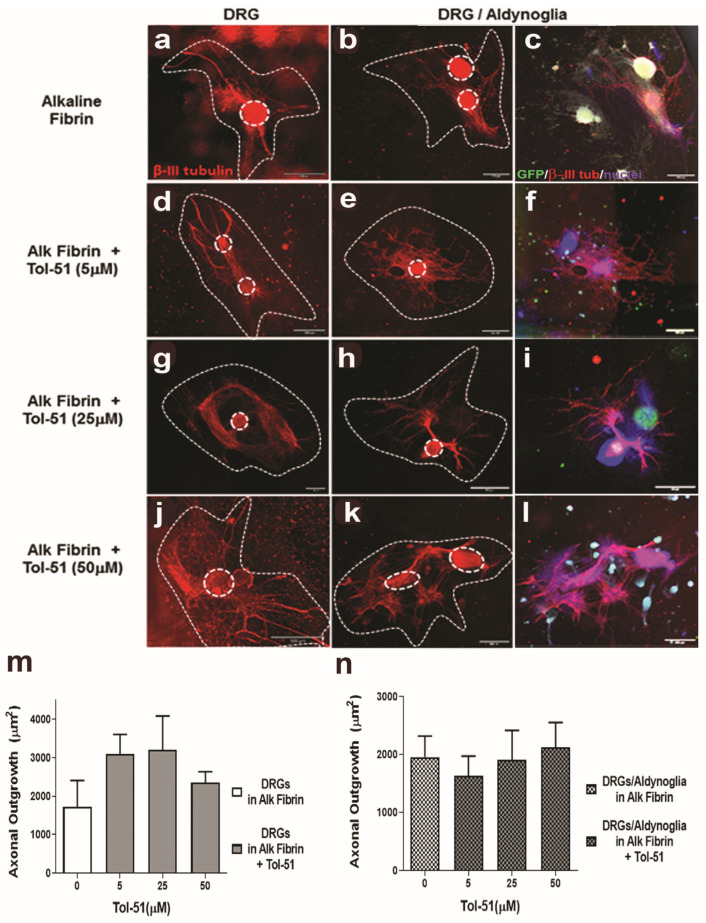
Alkaline fibrin and Tol-51 sulfoglycolipid during axonal growth promotion of spinal neurons by aldynoglia. DRGs were plated on alkaline fibrin containing aldynoglia cells and incubated in the presence of Tol-51 for ten days. Axonal outgrowth of spinal neurons was obtained after subtracting the central body of ganglia (dotted circles) from the area of total axonal outgrowth (dotted lines) revealed by monoclonal anti-BIII tubulin antibody, in red. Some promotion of DRG axonal growth by Tol-51 was observed in the absence of aldynoglia cells (**a**,**d**,**g**,**j** and bar graph **m**) and in their presence (**b**,**e**,**h**,**k** and bar graph **n**), but differences were not significant. The presence of aldynoglia is revealed by GFP expression in merged images (**c**,**f**,**i**,**l**). Magnification bars at 500 µm. DRGs, dorsal root ganglia.

DRGs were cultured in alkaline fibrin containing aldynoglia cells and incubated in the presence of Tol-51 for 10 days. The extent of the axonal growth area was revealed by the β-III tubulin marker. Axonal growth of DRGs in the presence of Tol-51 increased up to 25 μM, but non-significant differences were obtained with respect to the control DRGs. A similar promotion of axonal growth within the alkaline fibrin hydrogel and the presence of aldynoglia cells plus Tol-51 were obtained. A dose–response trend of Tol-51 in axonal growth was observed, but no significant differences were obtained. We then concluded that treatment with alkaline fibrin and sulfoglycolipid Tol-51 does not affect the promotion of axonal growth of spinal neurons by aldynoglia. At the same time, it offers cellular support and avoids inhibition by Rho GTPase activity. This paves the way for a new combinatorial therapy in traumatic spinal cord injury (Figure 6).

### 2.6. Axonal Fasciculation of Spinal Neurons within Alkaline Fibrin Is Promoted by Aldynoglia and Tol-51 Sulfoglycolipid

Axonal fasciculation is promoted by aldynoglia in two-dimensional cultures [12]. Here, we wanted to corroborate whether this property was maintained in three-dimensional cultures of alkaline fibrin and the presence of Tol-51 compound. After 10 days of cell culture for DRGs–aldynoglia in alkaline fibrin and sulfoglycolipid Tol-51 at different concentrations (0, 5, 25 and 50 μM) the co-cultures were fixed for immunocytochemistry. The presence of neurons and axon bundles was revealed by the β-III tubulin antibody and the caliber of fascicles was measured in fluorescent microscopic images, as per [22].

**Figure 7:**
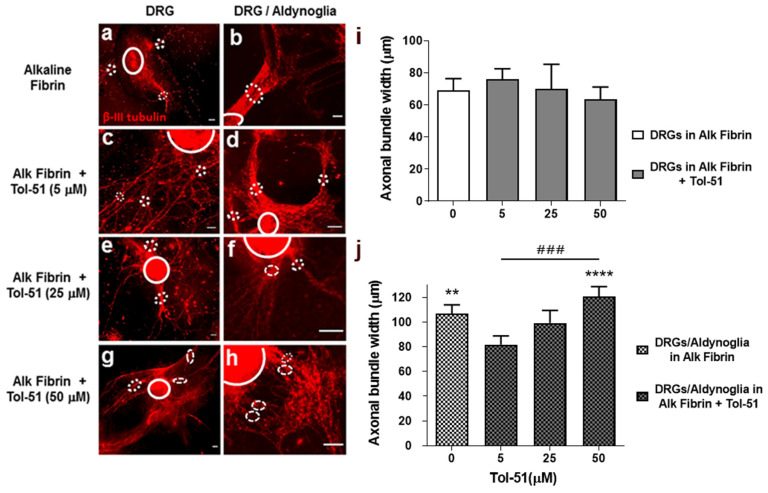
Alkaline fibrin and Tol-51 sulfoglycolipid promoted axonal fasciculation of spinal neurons by aldynoglia. DRGs were plated on alkaline fibrin containing aldynoglia cells and incubated in the presence of Tol-51 for ten days. Neurons and axonal bundles were revealed by monoclonal anti-βIII tubulin antibody, in red. The fasciculation of spinal neurons was obtained by measuring the axonal bundle width at ≥8.0 µm, dotted circles; and 20 µm away from DRG body, solid line. Axonal bundle fasciculation by Tol-51 similar to control was observed in the absence of aldynoglia cells (**a**,**c**,**e**,**g**; and bar graph in **i**). The aldynoglia presence increased axonal bundle width (**b**,**d**,**f**,**h**,**j**) and a significant dose response for assayed Tol-51 concentrations were obtained (###; *p* = 0.0006). Values of (**i**) were compared with counterparts in (**j**); ** *p* = 0.01; **** *p* = 0.0001; one-way ANOVA. DRGs, dorsal root ganglia; magnification bars at 500 µm.

We observed that axonal fasciculation of spinal neurons from DRGs within alkaline fibrin was not affected by increasing concentrations of sulfoglycolipid Tol-51 (Figure 7i). However, the presence of aldynoglia in the cell culture significantly promoted axonal fasciculation, as expected; *p* = 0.01 (Figure 7h). A significant increase in axonal fasciculation of DRG neurons was obtained by the addition of sulfoglycolipid Tol-51 to DRGs–aldynoglia co-cultures (*p* = 0.0001), and occurred in a dose-dependent manner with a significant difference, *p* = 0.0006 (Figure 7h). The Nogo A protein, which activates the RhoA/ROCK cascade, is present in DRGs and inhibits axonal fasciculation [23]. We then concluded that inactivation of RhoA/ROCK by Tol-51 had an additive effect on the axonal fasciculation of spinal neurons promoted by aldynoglia cells (Figure 7).

## 3. Discussion

In general, therapeutic approaches in traumatic spinal cord injury (tSCI) seek the modulation of selected extracellular, cellular, or molecular processes related to inflammation or glial scarring, as well as preserving spinal tissue not involved in the initial injury [24,25]. Here, we describe for the first time the simultaneous functional interaction of three different elements: alkaline fibrin hydrogel, aldynoglia cells, and sulfoglycolipid Tol-51. We and others have reported that these elements can independently improve recovery from tSCI [2,5,17,26].

Local inhibition of Rho GTPase activity at the site of injury has been linked to functional recovery after tSCI [15,25,27]. However, as far as we have been able to review, the influence of Rho GTPase inhibition and alkaline environment for cells with therapeutic potential on spinal regeneration after tSCI has not been published. Recovery after tSCI needs to improve/preserve crucial events in neural cell functions, such as cell migration, cell differentiation, promotion of axonal growth, and axonal fasciculation [22,28,29,30]. Here, we reveal that aldynoglia, alkaline fibrin, and Tol-51 elements under in vitro conditions can increase neural cell differentiation (Figure 3 and Figure 4) and neural cell migration (Figure 5), maintain axonal growth (Figure 6), and promote axonal fasciculation (Figure 7).

Scaffolds plus cell transplantation are more effective than scaffolds and cell therapy alone in improving locomotor function in the acute phase of injury after SCI when used in animal models [31]. A Bayesian network meta-analysis found that bone marrow stem cells (BMSCs) combined with scaffolds, such as fibrin, are more effective in improving motor functional recovery than BMSCs and scaffolds alone, and could be an option in regeneration therapy for patients with SCI [26].

Fibrin is a component of the extracellular matrix involved in wound healing and is often present in traumatic SCIs as part of blood clotting [32]. The transparency and laxity achieved in alkaline fibrin hydrogels are quite useful properties for monitoring cell migration and degradation of biomaterials used as a vehicle or substrate for cell transplantation (Figure 2 and Figure 3). It has been reported that acidic extracellular pH triggers NLRP3 inflammasome and interleukin 1β secretion in human macrophages, which constitutes a warning signal of innate immunity [33]. A basic extracellular pH due to the presence of alkaline fibrin could contribute to halt the innate immune response and at the same time favor the migration of neural cells in the transplant environment.

Aldynoglia cells are an excellent candidate for cell transplantation in traumatic SCI due to their intrinsic properties for cell ensheathment, promotion of axonal growth, and remyelination of denuded axons [12]. Individualized rodent GFP–aldynoglia cells showed later differentiation in three-dimensional cell cultures than in two-dimensional ones, as expected (Figure 3, Figure 4 and Figure 5). However, cell clustering was observed in GFP–aldynoglia cells before differentiation in alkaline fibrin hydrogels that did not occur in two-dimensional culture conditions, perhaps due to induction of cell migration by fibrin [34] or diffusible migrating factors secreted by NPCs [35]. In addition, long GFAP+ cellular processes, which eventually connect clusters of aldynoglia, were frequently observed (Figure 3 and Figure 4). These glial processes are involved in ensheathment, myelination of axons, fasciculation, and axonal outgrowth [12].

GFAP expression occurs during normal differentiation of neural precursor cells (NPCs) and glial scarring after tSCI [36,37,38]. It is important to know where the GFAP expression corresponds to normal NPC differentiation or experimental manipulation. We observed an increase in GFAP expression for GFP–aldynoglia cells by culture on alkaline fibrin variants compared to fibrin cultures at physiological pH (Figure 3). The addition of the sulfoglycolipid Tol-51 did not cause a significantly greater increase in GFAP expression than that obtained in the alkaline fibrin variants (Figure 4). In this regard, it has been reported that extravasation of fibrinogen into the cerebral cortex was significantly associated with an increasing degree of astrogliosis [39]. Therefore, the resulting increase in GFAP expression could be associated with the presence of unreacted fibrinogen that was not consumed during gelation of the alkaline fibrin hydrogel and produced some glial reactivity of aldynoglia cells, but the sulfoglycolipid Tol-51 did not appear to have an additional contribution to glial reactivity (Figure 4).

Cell migration and degradation of fibrin gels occurs simultaneously in 3D cell cultures of astroglial cells [10]. A dynamic cell interaction was also observed in DRG/GFP–aldynoglia co-cultures within alkaline fibrin hydrogels (Figure 5). Invasion of GFP–aldynoglia into DRGs, concomitant with degradation of the alkaline fibrin hydrogel by GFP–aldynoglia, and migration of neural cells are desired properties for efficient insertion of cell grafts, differentiation of neural cells, and better recovery of functions lost due to an injured CNS.

Cell adhesion molecules influence axonal growth, are responsible for axonal fasciculation, and also participate in regeneration after injury to the peripheral nervous system through the function of Schwann cells [40]. Aldynoglia cells derive from the striatum of the CNS and have similar properties to Schwann cells, with high expression of cell adhesion molecules such as intercellular adhesion molecule 1 and neuron glia-related cell adhesion molecule—ICAM1 and NrCAM, respectively [12]. ICAM1 and NrCAM participate in axonal growth and axonal fasciculation [41,42], which is consistent with promoting axonal growth and fasciculation of neurons co-cultured with aldynoglia cells (Figure 6 and Figure 7).

The sulfoglycolipid Tol-51, formerly IG20, sequesters Rho GDIα and produces an inhibition of Rho GTPase activity. The inhibition is caused by uncoupling of BDNF/TrkBT1/Rho GDIα/Rho GTPase signaling cascade that operates in astrocytes and microglia cells [15,17,43]. In two-dimensional cell cultures, Tol-51 promotes axonal outgrowth in DRG–aldynoglia co-cultures [17].

In DRG–aldynoglia co-cultures within alkaline fibrin, a non-significant positive effect for Tol-51 on axonal growth was obtained (Figure 6). We can infer that the sulfoglycolipid Tol-51 did not interfere with the promotion of axonal growth produced by aldynoglia, making Tol-51 a useful adjuvant against inhibitors of axonal growth, such as molecules with Rho GTPase activity after tSCI. Furthermore, at high doses of sulfoglycolipid Tol-51 in 3D alkaline fibrin co-cultures, there was a significant additive effect on axonal fasciculation and a dose-dependent effect was observed for the Tol-51 inhibitor (Figure 7). Myelin-derived proteins that activate the RhoA/ROCK cascade are present in DRGs and the tSCI lesion site and inhibit axonal fasciculation [23,44].

In the rat tSCI model of complete spinal cord transection, we injected Tol-51 sulfoglycolipid cells and aldynoglia into both spinal stumps. Next, we filled the cavity left by the resection of the fibroglial scar in the spinal cord with pre-degenerated peripheral tissue and sealed the graft area with alkaline fibrin. Under these conditions, we have some encouraging preliminary results in our ongoing experiments. Three months after transplantation, firm insertion of the graft into the injured spinal cord was observed (Supplementary Figure S1). The treated animals show significant functional recovery compared to the control group. We believe that the enhancement of axonal growth and axonal fasciculation by aldynoglia plus Tol-51 in the presence of alkaline fibrin supports these preliminary results and could constitute a combinatorial therapy in the treatment of tSCI.

## 4. Methods

### 4.1. Animals

All experiments followed European Council directive 2010/63/EU guidelines to limit pain and discomfort to experimental animals. The study was approved by the local Animal Welfare Ethical Committee of the National Hospital for Paraplegics (No. 48-OH/2021) and ratified by the Department of Agriculture, Water and Rural Development of the Government of Castilla La Mancha (No. 8-2021). In this study, we used E15 embryos from Sprague Dawley rats, strain SD-Tg(UBC-EGFP)2BalRrrc containing the enhanced green fluorescent protein gene under the control of the human ubiquitin C promoter (SD-GFP rats) and Wistar rats. The animals were bred and housed in the animal facility of the Paraplegics Hospital.

### 4.2. Neural Precursor Cells

Isolation of neural precursor cells and cell culture followed the procedures reported by us and others [12,45]. Briefly, E15 rat embryos were obtained by cesarean section from deeply anesthetized pregnant Wistar or SD-GFP rats. The striatum of E15 embryos was dissected and mechanically dissociated into single cells. Cells were seeded in a 75 cm^2^ culture flask with 15 mL of Neurobasal medium plus B27 supplement, called NB27 medium (Gibco, Madrid, Spain), containing 10 ng/mL human bFGF and 20 ng/mL human EGF (Peprotech, NJ, USA), 1% L-glutamate, 200 mM (Sigma, St. Louis, MO, USA), and 5000 U/mL penicillin–streptomycin (Gibco, Madrid, Spain). After a week in cell culture, floating neurospheres were obtained and collected by centrifugation, dissociated by mild trypsinization, and passed through a 25 mm needle. Neurospheres were dissociated and expanded every 3–4 days and used after the fourth passage in cell culture.

### 4.3. Synthesis and Detection of Tol-51 Compound

The organic synthesis of the sulfoglycolipid Tol-51 was performed following the instructions previously described in [19]. Identification of synthetic sulfoglycolipid at 550.3 (*m*/*z*) was monitored on the 4800 Plus MALDI TOF/TOF analyzer (Applied Biosystems, San Francisco, CA, USA), as previously reported [20].

### 4.4. Alkaline Fibrin

The two frozen components from Tisseel (Baxter, Spain), human fibrinogen (91 mg/mL) plus synthetic aprotinin (3000 IU/mL), and human thrombin (500 IU/mL) plus CaCl_2_ (40 µmol/mL) were gently thawed at room temperature and kept in an ice bath, then dispensed in separate aliquots and stored at −20 °C until use. To obtain alkaline fibrin, frozen aliquots of fibrinogen–aprotinin were gently thawed and diluted 1:1 with concentrated Tris buffer, TBS pH = 10. This mixture was diluted 2:1 with thrombin–CaCl_2_ for gelation of alkaline fibrin hydrogel. The final concentration of TBS in alkaline fibrin was Tris base 25 mM, NaCl 137 mM, and KCl 2.6 mM. After 1 h incubation at room temperature for normal or 2× alkaline gel reactions, a translucent alkaline fibrin hydrogel was obtained.

### 4.5. Cell Encapsulation in Alkaline Fibrin

Neural precursor cells (NPCs; 1 × 10^5^) were added to aliquots of fibrinogen–aprotinin to encapsulate them during alkaline fibrin gelation. The NPCs suspension was plated in 48-well plastic plates for cell culture (Falcon, NJ, USA), and thrombin–CaCl_2_ was added for gelation. The alkaline hydrogel containing NPCs was incubated with NB27 plus 10% inactivated bovine serum (Gibco, New Zealand) to facilitate cell differentiation to aldynoglia for two weeks, and the cell culture medium was changed every 3 days.

### 4.6. Immunocytochemistry

Treated cells on multiwell plates were fixed in 2% paraformaldehyde in PBS (12 min, 25 °C), washed with PBS, and immunostained as follows. They were incubated for 45 min at 25 °C in the presence of 0.1% Triton X-100 in PBS containing 1% normal goat serum. The cells were then incubated overnight at 4 °C in PBS containing the primary antibodies. We used an anti-GFAP rabbit polyclonal (Origene, USA) and anti-GFP mouse monoclonal (Invitrogen, Spain) to distinguish glial differentiated cells over total cell distribution, respectively, and for axonal growth, an anti-β-III tubulin rabbit polyclonal (Abcam, Spain) was used. All primary antibodies were assayed at 1/200 dilution. After repeated washing with PBS, the fixed cells were incubated with secondary antibodies (Alexa Fluor 594, goat anti-rabbit; Alexa Fluor 488 goat anti-mouse, Molecular Probes, Spain) for 2 h at 25 °C, all at a dilution of 1/500. After secondary antibody incubation, cells were incubated with 10 µg/mL Hoechst agent (Sigma Aldrich, St. Louis, MO, USA) in PBS for 10 min at 25 °C and washed three times with PBS. Images were acquired using phase-contrast and fluorescence microscopes: Leica DMI 6000B with 10× lens and Olympus IX83 4× with lens inverted.

### 4.7. Cell Differentiation and Glial Outgrowth Assay

Cell differentiation was followed by fluorescence density analysis for each specific marker detected, GFP or GFAP. Histograms were obtained by plotting the fluorescence intensity values contained in the bitmap area of the images. The intensity of fluorescence and the spatial scale were previously calibrated to establish the intensity values as the integrated intensity of each image. A binary mask was used to determine the area of glial outgrowth and discard unwanted particles. A lower outgrowth limit was set at 10 microns, and the growth-specific signal area was represented in pixels/mm^2^. Immunostained cells were examined on a Leica DMI 6000B inverted microscope using a 10× objective lens, processed with LASAF software Version 2.7.3.9723, and analyzed with ImageJ 1.53f51 (FIJI) software [46], and 20 fields were analyzed for each sample replicate.

### 4.8. Cell Migration in Hydrogel-Encapsulated DRG–Aldynoglia Cell Co-Cultures

One day earlier, individualized GFP-NPCs isolated from SD-GFP neurospheres (2 × 10^5^ cells/well) were seeded in 48-well culture plates (Falcon, NJ, USA) and incubated in NB27 plus bovine serum (10%) to facilitate differentiation of GFP-NPCs to GFP-aldynoglia. After 24 h, culture medium was aspirated and freshly dissected dorsal root ganglia (DRGs) from E15 Wistar rats were deposited [12]. Three DRGs were seeded in each well covered with GFP-aldynoglia cells and then encapsulated with fibrin or alkaline fibrin hydrogel. The DRG–GFP-aldynoglia cells encapsulated in fibrin hydrogels variants were cultured for 10 days in medium plus serum containing Tol-51 sulfoglycolipid (µM) or without compound as a control.

To record the migration and behavior of GFP-aldynoglia and DRG cells into fibrin hydrogels, time-lapse video was recorded with a Leica DMI 6000B inverted microscope using a 10× lens during the first 4 days of cell co-cultures. The parameters for the time-lapse video recording were fields of 560 µm and nine captures every 70 µm in height on the Z axis. The best-focused video was selected and processed with LASAF software. The cell culture media were changed every three days, and after the incubation period, the co-cultures were fixed and immunostained.

### 4.9. Axonal Outgrowth and Fasciculation in Hydrogel-Encapsulated DRG–Aldynoglia Co-Cultures

Immunofluorescence images of β-III tubulin-positive areas in fixed DRGs were measured for each ganglion using ImageJ 1.53F51 (FIJI) software [46]. Each treatment was replicated, and each replicate was assessed by two different measures for axonal growth from DRGs. Two regions of interest (ROIs) were generated, one ROI covering the entire area of axonal growth (both ganglion and neuritic halo), and the other ROI for the DRG body, where the soma of the neurons resides. After subtracting the ROI corresponding to DRG body area, we obtained the axonal outgrowing area.

In axonal fasciculation analysis, image captures were taken at different levels of the Z axis along the 3D shape of the fibrin hydrogel. The obtained Z-stack image was used to perform extended focus imaging (EFI) to transform the stack into a focused 2D image. Axonal fasciculation was measured by the width of the axon bundle in the resulting 2D image, considering diameters ≥ 8 µm [22]. An Olympus IX83 inverted microscope with a 4× lens was used for the analysis of fasciculation and axonal growth.

### 4.10. Statistical Analysis

Prior to any data analysis, a Pearson comparison was conducted to define normality for the statistical test to use. For cell differentiation analysis, we used unpaired t tests followed by parametric one-way ANOVA with post hoc multiple-comparison Tukey tests, using a significance level of *p* = 0.0001. In outgrowth assays, we used non-parametric one-way ANOVA with multiple comparisons by Kruskal–Wallis probes for all the groups, with significance set at *p* = 0.05. In fasciculation analysis, we used unpaired *t* tests followed by parametric one-way ANOVA with post hoc multiple-comparison Tukey tests for all the groups, with significance set at *p* = 0.05. All statistical analyses were performed using GraphPad Prism V9.0.1.

## 5. Conclusions

Traumatic SCI (tSCI) is a heterogeneous condition that causes loss of sensation and locomotor function with deterioration of neural tissue. Here, we attempted to observe and improve in vitro crucial neural functions (neural cell migration and differentiation, axonal growth and axonal fasciculation) using an alkaline fibrin hydrogel containing aldynoglia as neural precursor cells plus the administration of the sulfoglycolipid Tol-51, an inhibitor of astroglia and microglia cells. The beneficial properties of each independent element are not diminished by the presence of the others, supporting its use as a functional bridge to replace degenerated neural tissue and improve recovery after tSCI. This study should be validated in animal models to monitor three-element graft tolerance, morphology, and functional recovery from transplant surgery after tSCI. The platform of aldynoglia, alkaline fibrin and Tol-51 sulfoglycolipid constitutes a promising multifunctional approach for a combinatorial therapy in tSCI.

## Data Availability

Data are contained within the article and Supplementary Materials.

## Supplementary Materials

The following supporting information can be downloaded at: https://www.mdpi.com/article/10.3390/ijms25179173/s1.
