# Supplementary material for: Axonal Growth and Fasciculation of Spinal Neurons Promoted by Aldynoglia in Alkaline Fibrin Hydrogel: Influence of Tol-51 Sulfoglycolipid
Source: Int J Mol Sci. 2024 Aug 23;25(17):9173. doi: 10.3390/ijms25179173 (PMC11395328; doi:10.3390/ijms25179173)
Supplement: Supplementary file 1 [file ijms-25-09173-s001.zip › ijms-3111774-supplementary.pdf]

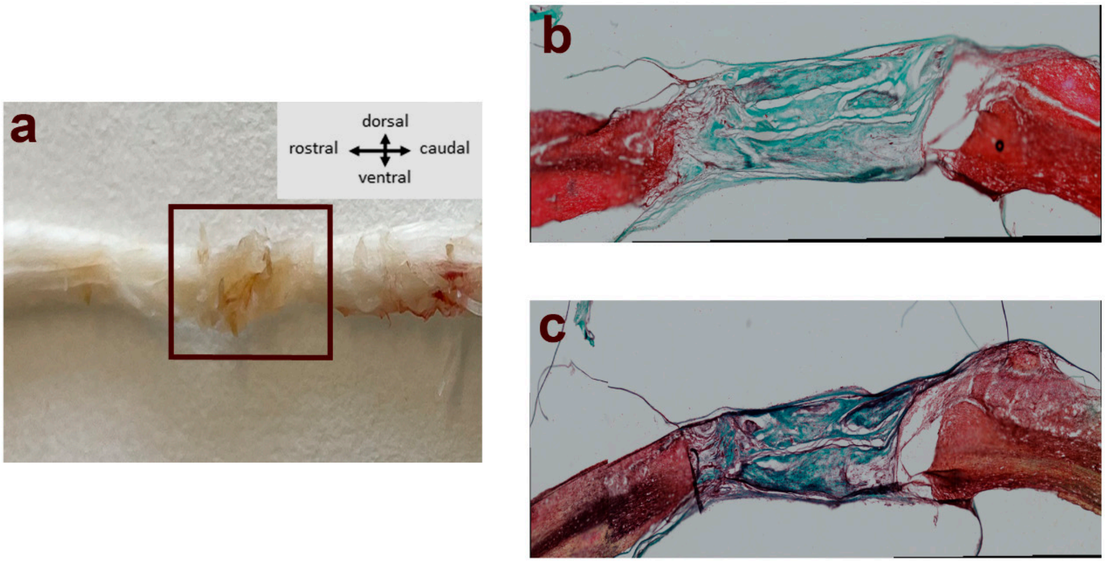

Figure S1. Transsected spinal cords with graft and treatment of alkaline fibrin, aldynoglia cells and sulfoglycolipid Tol-51. A macroscopic view of the transsected and transplanted spinal cord after 3 months of transplantation; graft orientation and open box for transplant zone are shown (a). Histological analysis of two other transsected and transplanted spinal cords, which were fixed and Masson stained for collagen fibers three months after transplantation (b, c). Note in all cases the firm insertion and continuity of the graft in spinal tissue of the host.
